# Anesthesia Management in a Crisponi Syndrome Patient Undergoing Tracheotomy Surgery

**DOI:** 10.1155/crpe/7470643

**Published:** 2025-07-11

**Authors:** Enes Celik, Yusuf Ipek, Osman Oguzhan Kursun, Mehmet Nur Talay, Hakan Akelma

**Affiliations:** ^1^Department of Anesthesiology and Reanimation, TC Mardin Artuklu Universitesi Tıp Fakultesi, Mardin, Turkey; ^2^Department of Anesthesiology, TC Saglık Bakanlıgı Mardin Egitim ve Arastırma Hastanesi, Mardin, Turkey; ^3^Department of Pediatrics, TC Mardin Artuklu Universitesi Tıp Fakultesi, Mardin, Turkey

**Keywords:** airway management, anesthesia, hyperthermia

## Abstract

Increased salivation and contractions of the oropharyngeal muscles are frequently observed in Crisponi syndrome. This causes frequent recurrent lung infections. Anesthesia management can be challenging due to the frequent convulsions that occur during the intubation and extubation of the patient and subsequent cyanosis and hyperthermia attacks. Cold-induced sweating attacks may also occur due to the low operating room temperature. Hyperthermia attacks can lead to rhabdomyolysis and disseminated intravascular coagulation. Sudden deaths may occur in children with Crisponi syndrome. Hyperthermia, paroxysmal muscular contractions and trismus due to autonomic dysfunction are held responsible for sudden deaths.

## 1. Introduction

Crisponi syndrome is an autosomal recessive disease that was first identified in the Sardinia region of Italy in 1996. Crisponi syndrome is characterized by the neonatal onset of episodes of marked muscular contraction of the facial muscles with trismus and excessive salivation simulating a tetanic spasm. Additional features include characteristic facial abnormalities, intermittent hyperthermia, feeding difficulties, and bilateral camptodactyly. Major feeding and respiratory difficulties with access of hyperthermia occur in the course of the disease and usually lead to death in the first months of life. In the rare survivors, hyperexcitability disappears in the first years of life and children develop psychomotor retardation [1]. Infants who survive over the first critical period gradually develop kyphoscoliosis, elevated plasma noradrenaline levels, and cold-induced sweating in their later years. In fact, this condition is characterized by profuse sweating induced not only by temperatures below 18°C–22°C (paradoxical sweating) but also by strong emotional stimuli or ingestion of sweets [2]. Anesthesia management is difficult due to increased salivation, skeletal deformities, and hyperthermia-convulsion attacks. We aimed to present the difficulties that may be encountered in anesthesia management with the help of literature.

## 2. Case Report

**Figure 1:**
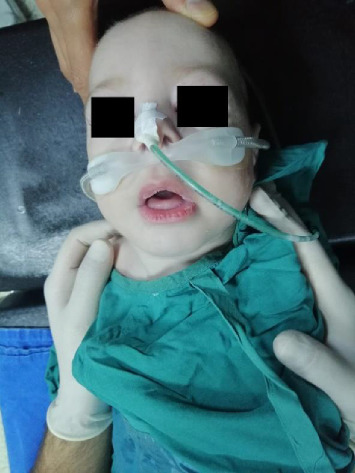
Face of a typical Crisponi syndrome patient, characterized by full cheeks.

Our case is a nine-month-old male patient (Figure 1). Elective tracheotomy operation was planned. Our patient was born by cesarean section at 39 weeks and weighing 3400 g. He was later taken to intensive care due to respiratory failure. The patient had a history of aspirating secretions and frequent lung infections. He was tachypneic with high flow oxygen support. Anticipating that the intubation process would be prolonged and to avoid complications of difficult intubation, elective tracheotomy was planned.

**Figure 2:**
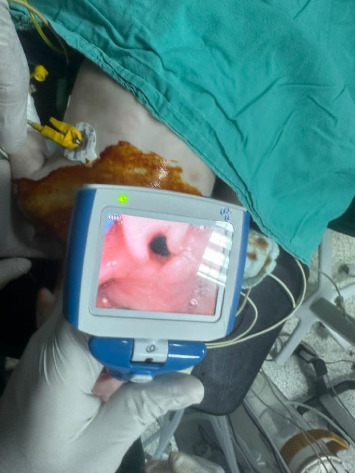
Edematous appearance of the airway and epiglottis on videolaryngoscopy.

In the family history, it was determined that the patient's parents were cousins. The family previously had a child who died when he was 9 months old, and Crisponi syndrome was diagnosed with genetic testing. The patient had bilateral camptodactyly in his hands. He was constantly in the opisthotonus position and had no sucking reflex. The patient was admitted to the operation room. Standard American Society of Anesthesiologists monitoring was performed. Anesthesia induction was achieved with 90% oxygen inhalation and 2.5 mg/kg propofol infusion. After effective mask ventilation, fentanyl (1.5 mcg/kg) and 0.5 mg/kg rocuronium were administered. Endotracheal intubation was performed on the first attempt with a 4.0-cuff endotracheal tube (ETT) using UEscope video laryngoscopy (Zhejiang UE MedicalCorp, Zheijang, China) (Figure 2). During videolaryngoscopy, it was observed that the epiglottis and airway were edematous, and the vocal cords were compatible with Cormack–Lehane 2. We noticed increased secretion in the mouth and pharynx.

Propofol infusion (6 mg/kg/hour) was started to maintain anesthesia. The surgery took approximately 35 min. At the end of the procedure, residual neuromuscular blockade was antagonized with sugammadex (2 mg/kg), and the patient was extubated. Postoperative analgesia was provided with paracetamol (10 mg/kg). No complications occurred. After surgery, the patient was safely transferred to the pediatric intensive care unit (Figure 2).

## 3. Discussion

Increased salivation and contractions of the oropharyngeal muscles are frequently observed in Crisponi syndrome. This causes frequent aspiration and recurrent lung infections. For these reasons, patients are operated on with the indication of percutaneous endoscopic gastrostomy (PEG) or tracheotomy. The extubation process may be prolonged in the postoperative period due to frequent lung infections of patients. In addition, anesthesia management can be challenging due to the frequent convulsions that occur during the intubation and extubation of the patient and subsequent cyanosis and hyperthermia attacks. Cold-induced sweating attacks may also occur due to the low operating room temperature.

Hyperthermia attacks have not been associated with anesthetic agents in the literature. Autonomic dysfunction is held responsible for hyperthermia attacks. It can also be confused with neonatal tetanus because convulsions are observed in the neonatal period [3]. Additionally, due to the increase in the frequency of facial hypoplasia and micrognathia in patients, difficulties may be experienced during bag mask ventilation and may make endotracheal intubation difficult. Difficult airway preparation should be made, including different sized masks, supraglottic airway devices, videolaryngoscopy, and videobronchoscopy.

The epiglottis may be edematous due to frequent contraction of the oropharyngeal muscles and recurrent upper airway infections. It should not be forgotten that intubation may be difficult due to airway edema and contraction position. In cases where airway edema has been previously detected, an intubation tube half a size smaller may be preferred.

Although it was not observed in our case, scoliosis may be observed in patients. This should not be ignored when positioning during the operation. We attribute the fact that we did not find any skeletal deformity in our case to the fact that our patient was less than 1 year. Generally scoliosis becomes evident after 1 year of age [1].

Sudden deaths may occur in children with Crisponi syndrome. Hyperthermia, paroxysmal muscular contractions and trismus due to autonomic dysfunction are held responsible for sudden deaths. Hyperthermia attacks, especially those that can reach 42°, can lead to rhabdomyolysis and disseminated intravascular coagulation [4]. The coldness of the operating room may trigger hyperthermia attacks. In particular, CLCF1 gene mutation is held responsible for cold-triggered hyperthermia attacks. Temperature monitoring can be performed to avoid serious consequences of hyperthermia attacks, including DIC and death. The operating room temperature should be kept at the highest possible values. Cooled isotonic crystalloid should be kept ready to be administered in case of possible hyperthermia attacks.

Crisponi syndrome can also be confused with Stuve–Wiedemann syndrome due to kyphoscoliosis, camptodactyly, feeding difficulties, and hyperthermia attacks. An increased frequency of difficult airway and malignant hyperthermia has been described in Stuve–Wiedemann syndrome [5]. In order to make anesthesia management safer, these syndromes should be differentiated before the operation. Genetic tests can be used for this purpose. CRLF 1 gene analysis may be helpful in distinguishing Crisponi syndrome.

There are few literature studies on general anesthesia management in patients with Crisponi syndrome. In our case, we used propofol infusion in general anesthesia management. And a single dose of rocuronium was enough. Sevoflurane was used in the studies of Allary et al. However, no muscle relaxant was used [4]. In Rafiq et al.'s study, sevoflurane and rocuronium were used. And neostigmine was used to reverse the effect of the muscle relaxant [1]. Another reason why we used propofol infusion in our case is that we were afraid that the patient might have other undiagnosed diseases. The fact that their parents were related and their previous history of child death made us uneasy about this issue. Detailed genetic analysis was not performed for autosomal recessive diseases caused by consanguineous marriage. By choosing to use sugammadex at the end of the operation, we aimed to protect against the increase in salivation caused by neostigmine. In Crisponi syndrome, there is both increased salivation and a defect in the swallowing reflex. Atropine can be used in practice to reduce the increase in salivation and parasympathetic activity caused by neostigmine. The second reason why we prefer sugammadex is to protect against cardiac side effects that atropine may cause.

Due to the frequent occurrence of consanguineous marriages in our country and region, we may encounter autosomal recessive diseases such as Crisponi syndrome. In the anesthetic approach, appropriate and constant room temperature should be ensured and difficult airway preparation should be made. Cooled isotonic crystalloid should be kept ready for hyperthermia attacks, and body temperature monitoring should be done. It should not be forgotten that respiratory problems may occur in the postoperative period due to recurrent lung infections, and an intensive care unit should be prepared to be used if necessary.

## Data Availability

The data that support the findings of this study are available from the corresponding author upon reasonable request.
